# The Influence of Patient–Provider Communication on Self‐Management Among Patients With Chronic Illness: A Systematic Mixed Studies Review

**DOI:** 10.1111/jan.16492

**Published:** 2024-09-28

**Authors:** Christin Iroegbu, Delphine S. Tuot, Lisa Lewis, Lea Ann Matura

**Affiliations:** ^1^ University of Pennsylvania School of Nursing Philadelphia Pennsylvania USA; ^2^ University of san Francisco School of Medicine San Francisco California USA; ^3^ Rutgers University–Camden Camden New Jersey USA

**Keywords:** chronic illness, patient self‐management, patient–provider communication, self‐care, self‐care management, self‐management

## Abstract

**Aim:**

To explore the influence of patient–provider communication on patient self‐management of chronic illness.

**Design:**

Systematic Mixed Studies Review.

**Data Sources:**

CINAHL, Google Scholar, EMBASE and PubMed were searched until March 2024.

**Methods:**

Employed a result‐based convergent design and the Mixed Method Appraisal Tool to evaluate studies. Narrative analysis, quantitative studies and thematic analysis for qualitative studies and overall results.

**Results:**

Thirteen articles published between 2003 and 2023 were included. Chronic illnesses studied: diabetes, heart failure, hypertension, chronic obstructive pulmonary disease and asthma. Data synthesis yielded the overarching theme: *adaptive interpersonal communication.* An approach that adapts communication content to each patient's unique needs, employs verbal and nonverbal communication, builds a connection and establishes patient rapport.

**Conclusion:**

Available evidence suggests that patient–provider communication influences chronic illness self‐management. A provider's ability to adjust and tailor their communication style is an important factor in helping patients to achieve optimal self‐management. Future research should explore this phenomenon in other common chronic illnesses not included in this review. Additionally, research on the patient's role in this process could help improve patient–provider communication.

**Implications for the Profession and/or Patient Care:**

Findings from this review have significant implications for shared and participatory decision making, where patients and providers collaborate to develop plans of care for patients to achieve optimal self‐management. Additionally, this review can contribute to the development of educational content and communication strategies for nurses and all healthcare professionals caring for patients with chronic illnesses.

**Impact:**

This is the first mixed studies systematic review to describe the influence patient–provider communication on patient self‐management of chronic illness. These findings consolidate existing evidence, providing a pathway for practical application to clinical practice and the potential to significantly impact the delivery of patient‐centred care and healthcare quality.

**Patient or Public Contribution:**

No patient or public contribution.

**Summary**

- What is already known?
  ○ Patient–provider communication plays a central role in disease management and the patient's understanding of their illness and treatment plan.
  ○ Despite advances in the treatment and management of chronic conditions, many patients with multiple chronic illnesses find monitoring, interpreting and managing their symptoms confusing.
  ○ Patients depend on providers to convey information clearly and effectively, minimising confusion and ensuring it is easily applicable to their daily lives.
- What this paper adds?
  ○ Consolidates the evidence of existing research and provides a comprehensive understanding of effective communication strategies.
  ○ Provides practical approaches for providers to enhance communication with all patients.
  ○ Identifies gaps in the current literature, such as the need for more research on various chronic illnesses, which can guide future research and expand our understanding of this topic.
- Implications for practice/policy?
  ○ Ongoing professional development training should include adaptive communication strategies that aid diverse patient needs and improve self‐management outcomes.
  ○ Policies should promote the adoption of patient‐centred care models that emphasise the importance of effective and tailored patient–provider communication.
  ○ Health systems and providers should prioritise creating a clinical environment that is supportive, empathetic and respectful of patient's needs and preferences for their treatment plans.

## 1 Introduction

Nearly half of the United States population—approximately 133 million Americans—suffer from at least one chronic illness. Chronic illness is defined as a condition that lasts longer than 1 year, requires ongoing medical care and/or limits activities of daily living (CDC 2022). Chronic illness is the main driver of healthcare costs—accounting for 90% of the $4.1 trillion spent on health care annually (CDC 2022; Ivynian, Newton, and DiGiacomo 2020). Management of chronic illness is a lifetime task for which patients are primarily responsible on a daily basis. However, it also requires the support and guidance from their healthcare providers, who offer vital information to assist patients in creating and maintaining an effective self‐management regimen. This reliance on providers for information is critical to helping patients achieve optimal health (Ivynian, Newton, and DiGiacomo 2020; Piette et al. 2003). Furthermore, there is an ongoing need to identify effective strategies and curriculum content to improve training methods and enhance the communication skills of healthcare providers (Burgener 2020).

Patient–provider communication plays a central role in disease management and the patient's understanding of their illness and treatment plan (Davis‐Ajami, Lu, and Wu 2022). To optimise patient self‐management, providers and nurses must not only empower patients but also ensure patients accrue the knowledge, confidence and skills to manage their illness (Piette et al. 2003; Wildevuur et al. 2017; Angwenyi et al. 2019; Byrne, Keogh, and Daly 2022). Despite our many advances in the treatment and management of chronic conditions, many patients with multiple chronic conditions find monitoring, interpreting and managing their symptoms confusing (Jin, Bratzke, and Baumann 2021; Riegel et al. 2021). This places significant reliance on the provider to effectively deliver information to patients in a way that minimises gaps in knowledge and confusion while also emphasising how to apply and incorporate this information to real‐life circumstances (Ivynian, Newton, and DiGiacomo 2020).

The purpose of this mixed studies review is to synthesise prior study findings on the influence of patient–provider communication on patient self‐management of their chronic illness. Findings from this review will serve as evidence to healthcare leaders and clinicians when making decisions for best practices and reducing variations in healthcare delivery (Gopalakrishnan and Ganeshkumar 2013). Chronic illnesses pose a significant global health challenge, affecting millions of people across all socioeconomic levels and geographic regions, while placing a substantial burden on healthcare systems worldwide. Elucidating the role of patient–provider communication in chronic illness self‐management contributes to our understanding of ways to improve patient–provider communication and enhance patient care experiences across healthcare settings on a domestic and global scale.

It is important to note that there are a variety of concepts and terms, some used interchangeably, to describe self‐management including but not limited to: self‐care, self‐monitoring, self‐care management, self‐care agency and self‐efficacy. While these may all be defined or described differently in the literature, there exists many commonalities between these concepts (Riegel et al. 2021). In this review, we define self‐management as the ‘ability of the individual, in conjunction with family, community, and healthcare professionals, to manage symptoms, treatments, lifestyle changes, and psychosocial, cultural and spiritual consequences of health conditions’ (Richard and Shea 2011). To focus this review, we limit the inclusion of articles to those that discuss the ability of the individual in conjunction with healthcare professionals to self‐manage and exclude articles that discuss patient self‐management in conjunction with family or community.

## 2 The Review

### 2.1 Aim

To explore the influence of patient–provider communication on patient self‐management of chronic illness.

## 3 Methods

### 3.1 Design

A result‐based convergent synthesis approach was employed to examine the nuances of patient–provider communication and its influence on patient self‐management of their chronic illness (Pluye and Hong 2014; Noyes et al. 2019). This design was chosen for its ability to analyse and synthesise quantitative and qualitative results separately and then combine and synthesise both results together to gain a more comprehensive understanding of the influence patient–provider communication has on patient self‐management (Noyes et al. 2019; Osokpo, James, and Riegel 2021).

### 3.2 Search Methods

A literature search was conducted in collaboration with a university librarian initially in March 2022 and updated in March 2024 in the following databases: CINAHL, EMBASE, Google Scholar and PubMed. Medical subject headings (MeSH), key terms and concepts related to patient–provider communication and self‐management were combined in the context of chronic illness and included the following search strategy: “communication”[Mesh] OR communication*[ti], “interpersonal relations”[Mesh] OR “Professional Patient Relations”) AND (“chronic disease”[mesh] OR “chronic disease*” OR "chronic illness*” OR “chronically ill” OR “chronically sick” OR “chronic sickness” OR “chronic condition*”) AND (“Self Care”[Mesh] OR “self‐care”[tiab] OR “self efficacy”[Mesh] OR “self efficacy”[tiab] OR “self management”.

## 4 Search Outcome

### 4.1 Inclusion and Exclusion Criteria

Articles were included if they were peer reviewed and presented, empirical quantitative data or original qualitative research that exclusively examined the relationship between patient–provider communication and patient self‐management, defined as the ability of the individual, in conjunction with healthcare professionals, to manage symptoms, treatments, lifestyle changes, and psychosocial, cultural and spiritual consequences of health conditions of a chronic illness. Articles that discussed the patient–provider relationship broadly, discussed the use of electronic communication or were of an opinion perspective were excluded. Additionally, articles that discussed self‐management outside of the context of patient–provider communication or patient self‐management in conjunction with family or community and not solely healthcare professionals were also excluded. There was no limit on publication date, and all articles included were published in English; articles without full text availability were also excluded.

## 5 Quality Appraisal

The quality of each study was appraised using the Mixed Methods Appraisal Tool (Pluye and Hong 2014; Hong, Gonzalez‐Reyes, and Pluye 2018). This tool is designed for methodological quality appraisal of systematic reviews. The author of this tool discourages the use of a total quality score and instead recommends the use of descriptive quality appraisal (Pluye and Hong 2014; Hong, Gonzalez‐Reyes, and Pluye 2018; Pluye 2013). Therefore, the studies included in this review were appraised based on a set of screening questions specific to qualitative mixed methods or quantitative study designs. Articles that met the criteria set by each appraisal question were considered high quality, and articles that did not meet criteria set by each appraisal question were considered to be of low methodological quality. Tables 1, 2, 3 list the appraisal questions and details of whether each article met the criteria or not with reasoning. All quantitative studies (see Table 1) and seven of the qualitative studies included in this review were appraised to have high methodological quality (see Table 2). The remaining qualitative study included in this review had a low methodological quality due to its qualitative approach used to answer the research question, the inadequacy of the data analysis methods used and the lack of coherency between the qualitative data source, collection, analysis and interpretation (see Table 2). The one mixed methods study included in this review is also of low methodological quality due to its lack of rationale for using a mixed methods design and lack of integration of qualitative and quantitative data (see Table 3).

**TABLE 1:** Mixed methods appraisal tool—quantitative studies.

Author/year	Are there clear research questions?	Do the collected data allow to address the research questions?	Is the sampling strategy relevant to address the research question?	Is the sample representative of the target population?	Are the measurements appropriate?	Is the risk of nonresponse bias low? OR are the confounders accounted for in the design and analysis?	Is statistical analysis appropriate to answer the research question? OR during the study period, is the intervention administered (or exposure occurred) as intended?
Piette et al. (2003)	Yes	Yes	Yes, a clear justification for the sample frame is provided and the sampling procedure is adequate	Yes, there is a clear description of the target population and of the sample, eligibility criteria are presented and eligible individuals that chose not to participate are described	Yes, the variables are clearly defined and accurately measured. The measures are justified and appropriate for answering each research questions	Yes, the variables are clearly defined and accurately measured, the measurements are justified and appropriate for answering the research question; the measurements reflect what they are supposed to measure; validated and reliability tested measures of the outcome of interest are used	Yes, statistical analyses used are clearly stated and justified
Baker et al. (2005)	Yes	Yes	Yes, a clear justification of the sample frame used is provided	Yes, a clear description of the target population and of the sample is provided	Yes, the variables are clearly defined and accurately measured, the measurements are justified and appropriate for answering the research question; validated and reliability tested measures of the outcome of interest are used	Yes, the variables are clearly defined and accurately measured, the measurements are justified and appropriate for answering the research question; the measurements reflect what they are supposed to measure; validated and reliability tested measures of the outcome of interest are used	Yes, statistical analyses used are clearly stated and justified
Heisler et al. (2007)	Yes	Yes	Yes, the source of sample is relevant to the target population and the sampling procedure is adequate	Yes, a clear description of the target population and of the sample is provided	Yes, the variables are clearly defined and accurately measured, the measurements are justified and appropriate for answering the research question; the measurements reflect what they are supposed to measure	Yes, appropriate methods to control for confounders are used	Yes, statistical analyses used are clearly stated and justified
Świątoniowska‐Lonc et al. (2020)	Yes	Yes	Yes, the source of sample is relevant to the target population and the sampling procedure is adequate	Yes, a clear description of the target population and of the sample is provided	Yes, the variables are clearly defined and accurately measured; the measurements are justified and appropriate for answering the research question; the measurements reflect what they are supposed to measure	Yes, the variables are clearly defined and accurately measured; the measurements are justified and appropriate for answering the research question; the measurements reflect what they are supposed to measure; validated and reliability tested measures of the outcome of interest are used	Yes, statistical analyses used are clearly stated

**TABLE 2:** Mixed methods appraisal—qualitative studies.

Author/year	Is the qualitative approach appropriate to answer the research question?	Are the qualitative data collection methods appropriate to address the research question?	Are the findings adequately derived from the data?	Is the interpretation of results sufficiently substantiated by data?	Is there coherence between qualitative data sources, collection, analysis and interpretation?
Ivynian, Newton, and DiGiacomo (2020)	Yes, the qualitative approach used was appropriate for the research question and problem	Yes, the method of data collection and the form of the data are adequate. Clear justifications is provided when data collection methods were modified during the study	Yes, data analysis methods used were appropriate for qualitative approach	Yes, the quotes provided to justify the themes is adequate	Yes, there are clear links between data sources, collection, analysis and interpretation
Peltola, Isotalus, and Åstedt‐Kurki (2018)	Yes, the qualitative approach was appropriate for the research question and problem	Yes, the method of data collection and the form of the data are adequate. Clear justification of both data collection methods (open ended e‐surveys and semistructured interviews) was presented	Yes, data analysis methods used were appropriate for qualitative approach	Yes, the interpretation of results was supported by the data collected	Yes, there were clear links between data sources, collection, analysis and interpretation
Kruse et al. (2013)	No, the qualitative approach (grounded theory) used was not appropriate for the research question and problem. For this study, ethnography may have been a more appropriate approach/method	Yes, the method of data collection and the form of the data are adequate to answer the research question. Although there was mention of direct observation, there appears only to be audio recording and no video recording or in‐person observation or memos noted	No, the data analysis method used was narrative analysis; however, what is described in the text is coding and theme development, which may align more with thematic analysis	Yes, the interpretation and the quotes provided to justify the themes were adequate	No, there were clear links between data sources, collection, analysis and interpretation. There were discrepancies between the approach, data collection and data analysis
Visse et al. (2010)	Yes, the qualitative approach (case study) used was appropriate for the research question and problem	Yes, the method of data collection and the form of the data are adequate. Clear justifications were provided when data collection methods were modified during the study	Yes, data analysis methods used were appropriate for a case‐study approach	Yes, the interpretation of results was supported by the data collected	Yes, there were clear links between data sources, collection, analysis and interpretation
Newcomb et al. (2010)	Yes, although the qualitative approach used was clearly stated, it was consistent with a qualitative descriptive approach and was appropriate for the research question and problem	Yes, the method of data collection (semistructured interviews developed from questionnaire) and the form of the data were adequate	Yes, data analysis methods used were appropriate for a qualitative descriptive approach	Yes, the interpretation of results was supported by the data collected. The quotes provided justify the themes and were adequate	Yes, there were clear links between data sources, collection, analysis and interpretation
Kumar (2007)	Yes, the qualitative approach used was appropriate for the research question and problem	No, data collection methods were not presented in this study	Yes, data analysis methods used were appropriate for a case‐study approach	Yes, the interpretation of results was supported by the theory used in this case study	Yes, there were clear links between analysis and interpretation. Data collection and sources were not revealed in this study
Jowsey, Gillespie, and Aspin (2011)	Yes, the qualitative approach was appropriate for research questions and problems	Yes, the method of data collection and the form of the data were adequate. Clear justification of both data collection methods (semistructured interviews) was presented	Yes, data analysis methods used were appropriate for qualitative approach	Yes, the data and quotes provided to justify the content presented were adequate	Yes, there were clear links between data sources, collection, analysis and interpretation
Kirk et al. (2023)	Yes, the qualitative approach was appropriate for research questions and problems	Yes, the method of data collection and the form of the data were adequate. Clear justification of both data collection methods (semistructured interviews/focus groups) was presented	Yes, data analysis methods used were appropriate for qualitative approach	Yes, the data and quotes provided to justify that the content presented was adequate	Yes, there were clear links between data sources, collection, analysis and interpretation

**TABLE 3:** Mixed methods appraisal tool—mixed methods studies.

Author/year	Is there an adequate rationale for using a mixed methods design to address the research question?	Are the different components of the study effectively integrated to answer the research question?	Are the outputs of the integration of qualitative and quantitative components adequately interpreted?	Are divergences and inconsistencies between quantitative and qualitative results adequately addressed?	Do the different components of the study adhere to the quality criteria of each tradition of the methods involved?
Claramita et al. (2020)	No, the reasons for conducting a mixed methods study were not clearly explained	No, there was no information on how qualitative and quantitative phases, results and data were integrated. Neither was there a discussion of how data gathered by both research methods was brought together to form a complete summary or when integration occurred. Results of the quantitative and qualitative studies were presented individually	No, there were no overall interpretations derived from integrating qualitative and quantitative findings as the qualitative and quantitative studies were presented separately	No, findings from the qualitative and quantitative components were not integrated, therefore divergence or inconsistencies were not addressed	Yes, Quantitative: clear description of the target population and of the sample (inclusion and exclusion criteria) reasons why certain eligible individuals chose not to participate was presented. The measurements reflect what they were supposed to measure and were justified and appropriate for answering the research question. The variables were clearly defined and accurately measured. Most participants were represented all measures. Confounding bias was low. During the study period, the intervention was administered as intended Qualitative: The qualitative approach was not clearly defined, but appeared to be qualitative descriptive, and this is appropriate to answer the research question. Data collection methods, including data sources used to address the research question, were adequate. Data analysis methods were consistent with the qualitative approach. The interpretation of results was supported by the data collected and quotes provided. There were clear links between data sources, collection, analysis and interpretation

## 6 Data Abstraction

The primary author worked independently to extract the following data from the included studies: author and year of publication; the type of chronic illness under investigation; country where the study was conducted; study aim; design, methods and sample characteristics; outcomes of patient–provider communication on the patient's ability in conjunction healthcare professionals, to manage symptoms, treatments, lifestyle changes, and psychosocial, cultural and spiritual consequences of health conditions of their chronic illness; and the strengths and limitations of the study (Table 4). A second reviewer confirmed the appropriateness of each article as defined by the inclusion/exclusion criteria and to minimise risk of bias.

**TABLE 4:** Mixed studies review table of evidence.

Author, year, title	Chronic illness	Study country	Study aims	Design/methods	Sample demographics	Study outcomes
Ivynian et al. (2020). Patient preferences for heart failure education and perceptions of patient–provider communication	Heart failure	Australia	To explore perceptions of patient–provider communication and ascertain unmet educational needs and preferences	Qualitative semistructured in‐depth interviews Thematic analysis	*Providers* Nurses Physicians The number of providers nor their demographic details were not reported *Patients* *N* = 15 9 Males 14 Females Median age: 55	Poor communication left patients without an understanding of how to identify and manage symptoms
Claramita et al. (2020). A partnership‐oriented and culturally sensitive communication style of doctors can impact the health outcomes of patients with chronic illnesses in Indonesia	T2DM and HTN	Indonesia	To test the effect of a communication skills training programme for doctors on patient's perception of physician's communication skills, physician's assessment of their communication skills, patient's health outcomes (blood pressure and glucose levels)	Mixed Methods Longitudinal Focus Groups Surveys Paired t‐tests Content Analysis Deductive Open coding	*Providers* *N* = 30 Primary care physicians 16 Males 19 Females Age range: 32–51 5–20 years of experience Race/ethnicity of providers not reported *Patients* *N* = 96 Patients *n* = 51 with T2DM *n* = 45 with HTN Age and race/ethnicity not reported	Patients' blood pressure or fasting blood glucose levels decreased significantly (*p* < 0.05), except the two‐hour blood glucose levels (NS) Patients expressed more satisfaction, increased comprehension and self‐management of their chronic illnesses
Peltola et al. (2018). Patients' Interpersonal Communication Experiences in the Context of Type 2 Diabetes Care	T2DM	Finland	To determine the relational communication characteristics of professional–patient communication situations that have either facilitated or impeded patients' self‐management	Qualitative Open‐ended e‐surveys and semistructured interviews Descriptive qualitative analysis	*Providers* Physicians Nurses The number of providers nor their demographic details were not reported *Patients* *N* = 16 Age range: 30–93 Gender: 13 Female 3 Males Race/ethnicity not reported	Both positive and negative experiences described by patients were connected to four multidimensional relational communication characteristics: (a) building trust in the other party in the professional–patient relationship, (b) willingness to communicate, (c) emotional presence and (d) appropriateness Portrayal of trust/distrust can impact patient's ability to self‐manage their DM Communicating an emotional presence can help patients understand the seriousness of managing their DM Showing respect and understanding motivated patients to improve self‐management behaviours
Kruse et al. (2013). Communication during patient–provider encounters regarding diabetes self‐management	T2DM	The United States	To provide an in‐depth analysis of how conversations of self‐care emerge during clinical encounters	Qualitative Direct observations Grounded theory Narrative analysis	*Providers* Nurse practitioner Physician Nurse partner The number of providers nor their demographic details were not reported *Patients* *N* = 30 Age range: 32–84 19 Females 11 Males Race/ethnicity not reported	Clinicians often focused their communication on quantitative measures such as blood pressure and glycosylated haemoglobin, but patients found it difficult to relate these measures to how they were feeling physically. Patients' social contexts influenced their self‐management activities. Supporting self‐management of patients with diabetes requires providers to link clinical measurements to patients' symptoms and likely outcomes. It is difficult for providers to know what support or assistance their patients need without knowledge of patients' social contexts
Visse et al. (2010). Dialogue for air, air for dialogue: towards shared responsibilities in COPD practice	COPD	The Netherlands	To examine the possibilities to enrich the notion of self‐management in a dialectical circle between practical understandings and theoretical insights from ethics	Qualitative Case study Interviews (open and semistructured) Narrative analysis	*Providers* Physicians (pulmonologist, *n* = 1) Therapists (lung physiotherapist, *n* = 2) The demographic details were not reported *Patient* *N* = 1 Male Age: not reported Race/ethnicity not reported	Patient–provider communication is a dialogical process that involves the provider exhibiting empathy, listening and understanding of the patient and the patient being able to share their illness as a story. Providers and patients are then able to engage in meaningful conversations where providers can provide practical support, advice and solutions for patients to manage their chronic illness
Newcomb et al. (2010). Barriers to patient–clinician collaboration in asthma management: the patient experience	Asthma	The United States	To describe what adult patients with asthma report about their experiences with their own self‐management behaviour and working with their clinicians to control asthma	Qualitative Semistructured interviews Observational field notes Open and selective coding Grounded theory Post hoc analysis Likert scale evaluation	*Providers* Primary Care Clinicians in general medicine clinics of an academic medical centre The number of providers nor their demographic details were not reported *Patients* *N* = 104 95 Females 9 Males Age mean: 50.0 Race: Asian *n* = 21 Black *n* = 25 White *n* = 83 Pacific Islander *n* = 1 Multiracial *n* = 9	These findings emphasise the difficulties of establishing and maintaining a therapeutic partnership between patients and clinicians. The results underscore the need for system‐wide interventions that promote the success of a therapeutic patient–clinician relationship in order to achieve long‐term success in chronic disease management
Kumar (2007). Application of Orem's self‐care deficit theory and standardised nursing languages in a case study of a woman with diabetes	T2DM	The United States	To illustrate the process of theory‐based nursing practice by presenting a case study of a clinical nurse specialist's assessment and care of a woman with type 2 diabetes	Qualitative Case study	*Provider* Nurse (*n* = 1) Physician (*n* = 1) The demographic details were not reported *Patient* *N* = 1 Age: 49 Female Race: White	Nursing theory and standardised nursing language enhance communication among nurses and support a client's ability to self‐manage a chronic illness
Piette et al. (2003). Dimensions of patient–provider communication and diabetes self‐care in an ethnically diverse population	Diabetes mellitus	The United States	To examine the impact of general versus disease‐specific communication on self‐management of diabetes among an ethnically diverse population within 3 separate systems of care	Quantitative Telephone interview surveys Interpersonal Processes of Care questionnaire Summary of Diabetes Self‐care Activities questionnaire (modified) Predicted probability models	*Providers* *N* = 328 Primary Care Providers in Veteran Administration health centres The demographic details were not reported *Patients* *N* = 848 Age ranges: < 50– +60 Males *n* = 30–105 Females *n* = 144–296 Race: White: 19%–72% Black: 13%–30% Hispanic: 4%–29% Other: 10%–29%	General and diabetes‐specific communication are independently related to improving DM self‐management. Providers on these sites are communicating successfully with vulnerable patients
Baker et al. (2005). A telephone survey to measure communication, education, self‐management and health status for patients with heart failure: the Improving Chronic Illness Care Evaluation (ICICE)education	Heart failure	The United States	To measure the influence of patient–provider communication, patient satisfaction, patient education, knowledge and self‐efficacy on patient self‐management and health status	Quantitative Heart Failure Symptom Scale Short form (SF)‐12 Physician Component Summary (PCS) Mental Component Summary (MCS) scale Consumer Assessment of Health Plans study instrument Communication Scale Knowledge of Heart Failure Physician and Nurse satisfaction scales Self‐efficacy scale	*Provider* Physician (*n* not reported) Nurse (*n* not reported) The number of providers nor their demographic details were not reported *Patients* *N* = 781 Age range: > 65 (62%) 375 Males 406 Females Race: White: 71% Other races not reported	Mean communication score 4.0 (range 1–5) 76% of patients reported working with a provider on a self‐management plan 66% of patients reported their physician explained things and 79% reported they listened carefully A supplemental written self‐management plan improved patient knowledge about heart failure Only 13% of participants knew how to effectively manage their heart failure independently Even with a high communication score, self‐management strategies were poorly understood by patients
Heisler et al. (2007). Does physician communication influence older patients' diabetes self‐management and glycemic control? Results from the Health and Retirement Study (HRS)	Diabetes mellitus	The United States	To assess the relative importance of two dimensions of physician communication provision of information (PCOM) and participatory decision making (PDM) for older patients' diabetes self‐management and glycaemic control	Quantitative Cross‐sectional Patient Assessment of Chronic Illness Care (PACIC) scale Provision of Information (PCOM) scale Haemoglobin A1C	*Provider* Physician The number of providers nor their demographic details were not reported *Patients* *N* = 1558 Age mean: 69 46% Male Race: White: 70% Black: 18% Hispanic: 10% Other: 2%	Among these older adults, both their diabetes providers' provision of information and efforts to actively involve them in treatment decision making were associated with better overall diabetes self‐management. PCOM and PDM were each associated with overall diabetes self‐management and with all self‐management
Świątoniowska‐Lonc et al. (2020). Impact of satisfaction with physician‐patient communication on self‐care and adherence in patients with hypertension: cross‐sectional study	HTN	Europe	To evaluate the relationship between physician–patient communication and self‐care and adherence in patient with hypertension undergoing chronic treatment	Quantitative Cross‐sectional Communication Assessment Tool (CAT) The Adherence to Refills and Medication Scale (ARMS) The Self‐Care of Hypertension Inventory (SCHI)	*Providers* Internal medicine with specialisation in hypertension The number of providers nor their demographic details were not reported *Patients* *N* = 250 Age mean: 61 110 Males 140 Females Race/ethnicity not reported	Satisfaction with physician–patient communication had a significant impact on self‐care and pharmaceutical adherence in patients with hypertension. The more satisfied the patient was with communication, the better their adherence and self‐care
Jowsey et al. (2011). Effective communication is crucial to self‐management: The experiences of immigrants to Australia living with diabetes	Diabetes mellitus	Australia	To describe the experiences of immigrants in Australia with type II diabetes mellitus (DM). What impact, if any, have health policies had on the lives of immigrants? How do their experiences of living with DM compare with those of people with DM who were born in Australia?	Qualitative Semistructured interviews Content analysis	*Provider* The provider type, number of providers, nor their demographic details were not reported *Patients* *N* = 34 Age range: 38–84 16 Males 18 Females Race: not reported Immigrants: *n* = 15 Australian born *n* = 19	Immigrants to Australia confront linguistic and cultural barriers that create an extra layer of problems not experienced by Australian‐born people. Older people who were born overseas face obstacles to effective engagement with the health system that weaken their ability to take an active part in the management of their conditions
Kirk et al. (2023). Exploring facilitators and barriers to patient‐provider communication regarding diabetes self‐management	Diabetes mellitus	The United States	To explore communication facilitators and barriers to diabetes‐specific communication in West Virginia	Qualitative Semistructured interviews Thematic analysis	*Provider* The provider type, number of providers, nor their demographic details were not reported *Patients* *N* = 57 Age mean: 62.1 17 Males 25 Females Race: White *n* = 40 Hispanic: *n* = 1 Black: *n* = 1 Other: *n* = 15	Three facilitators to patient–provider communication emerged: patient–provider partnership, provider accessibility and empowerment through education. Partnership with providers, who were accessible outside of scheduled appointments, and empowerment obtained through diabetes education facilitated. Diabetes‐specific patient–provider communication. Barriers included: providers' focus on ‘numbers’ rather than patient concerns, patient lack of preparation for appointments and providers ‘talking down to’ patients

*Note*: Sample demographics are reported as in the original article.

Abbreviations: COPD, chronic obstructive pulmonary disease; HTN, hypertension; T2DM, type 2 diabetes mellitus.

## 7 Synthesis

Quantitative data were synthesised narratively (Hong, Gonzalez‐Reyes, and Pluye 2018). Due to the variability in methodological design and the limited number of quantitative studies included in this review, a meta‐analysis was not performed. Qualitative studies were synthesised using thematic analysis and inserted into a matrix table (Hong, Gonzalez‐Reyes, and Pluye 2018; Thomas and Harden 2008). This was done using a three‐step approach: first, coding the text line by line for each study, second, developing descriptive themes from each study that reflected the aims, and third, generating analytical themes that reflect and interpret the constructs of patient–provider communication and patient self‐management. Data included in the matrix were reviewed iteratively and codes were generated (Hong, Gonzalez‐Reyes, and Pluye 2018). Generated codes were then grouped into categories to reveal emerging themes (Pluye and Hong 2014; Noyes et al. 2019; Thomas and Harden 2008). Following the analysis of quantitative and qualitative data, a combined synthesis of both data was performed to derive overarching themes from the integration of the data (Osokpo, James, and Riegel 2021).

## 8 Results

**FIGURE 1:**
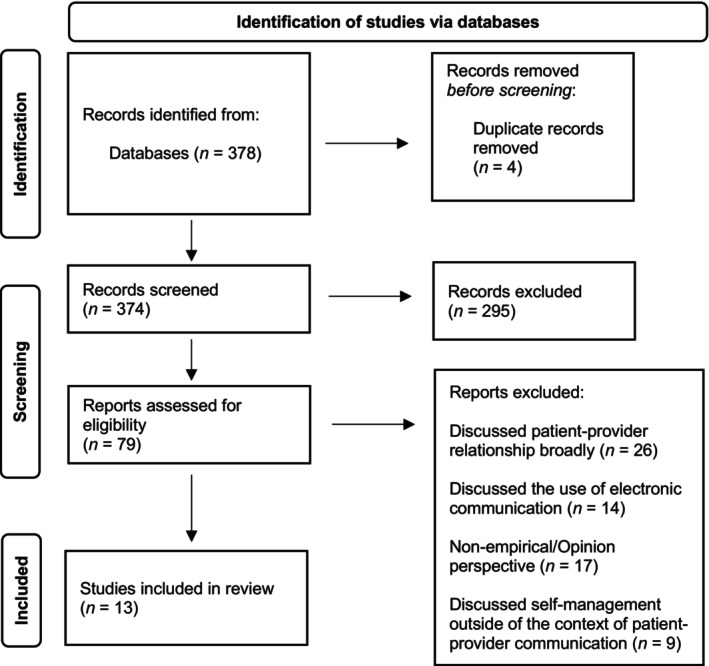
PRISMA diagram: Selection process of included articles.

A total of 378 articles were identified from PubMed, Google Scholar, EMBASE and CINAHL. Four duplicates were removed, and a total of 295 were excluded in title screening leaving a total of 79 articles for abstract screening. A total of 66 articles were excluded after abstract screening resulting in a total of 13, all of which are included in this review (Figure 1).

## 9 Characteristics of the Studies

Studies included in this review were qualitative (*n* = 8), quantitative (*n* = 4) and mixed methods (*n* = 1). All studies were published between 2003 and 2023 and were conducted in North America (*n* = 7), Europe (*n* = 5) and southeast Asia (*n* = 1). Diabetes was the most frequent chronic illness studied and the primary focus of seven studies (Piette et al. 2003; Peltola, Isotalus, and Åstedt‐Kurki 2018; Kruse et al. 2013; Kumar 2007; Heisler et al. 2007; Jowsey, Gillespie, and Aspin 2011; Kirk et al. 2023). Other chronic illnesses included heart failure, (Ivynian, Newton, and DiGiacomo 2020; Baker et al. 2005) hypertension, (Świątoniowska‐Lonc et al. 2020) COPD, (Visse et al. 2010) and asthma (Newcomb et al. 2010); one study investigated diabetes and hypertension together (Claramita et al. 2020).

Data were collected through surveys, individual interviews, observations, focus groups and electronic medical record review (Tables 4 and 2). All studies included adult men and women, but not all studies reported on race and ethnicity. Grounded theory, thematic, narrative, qualitative descriptive and qualitative content analysis methods were used in the qualitative studies. Quantitative studies used cross‐sectional analyses or survey results to describe the data and study outcomes. Sample sizes ranged from 1 to 104 in the qualitative studies and 848–1558 in quantitative studies.

Most studies sought to characterise the patient–provider communication encounter by describing the communication encounter, acquiring patient perceptions of their providers communication, or describe specific elements of the patient–provider clinical encounter (Ivynian, Newton, and DiGiacomo 2020; Peltola, Isotalus, and Åstedt‐Kurki 2018; Kruse et al. 2013; Kumar 2007; Heisler et al. 2007; Kirk et al. 2023; Newcomb et al. 2010). All studies explored or examined how these factors impeded, influenced or improved the patient's ability to manage their chronic illness. Self‐management of chronic illness was described or specified in the included studies as performing specific self‐care behaviours, measurement of a specific health outcome (ex. Haemoglobin A1c) or by a survey instrument score.

## 10 Synthesis of Quantitative Studies

Overall, quantitative studies found patient–provider communication to be influential on patient self‐management of a chronic illness. The quantitative studies included in this review focused on measuring the influence of patient–provider communication on key health outcomes. Outcomes measured for diabetes self‐management were foot care, medication adherence, diet and exercise (Piette et al. 2003; Peltola, Isotalus, and Åstedt‐Kurki 2018; Kruse et al. 2013; Kumar 2007; Heisler et al. 2007; Claramita et al. 2020). Outcomes measured for heart failure and hypertension included daily weight monitoring, consuming a low‐salt diet, seeking medical attention when appropriate and medication adherence (Baker et al. 2005; Świątoniowska‐Lonc et al. 2020). Tools used to measure provider communication were the Communication Assessment Tool (CAT), the Interpersonal Processes of Care (IPC) questionnaire, a previously unpublished diabetes‐specific communication scale, the Consumer Assessment of Health Plans Study instrument, the Patient Assessment of Chronic Illness Care (PACIC) scale and the American Board of Internal Medicine patient survey instrument.

Providers that embodied characteristics of a participatory communication style were associated with better patient self‐management of their chronic illness (Piette et al. 2003; Heisler et al. 2007). Providers that offered disease‐specific information tailored to patients' chronic illness were most influential on patients self‐managing their chronic illness (Piette et al. 2003; Heisler et al. 2007). Patients that worked with their provider in a collaborative fashion found it easier to self‐manage their chronic illness independently and effectively (Heisler et al. 2007; Baker et al. 2005). However, providers that did not deliver disease‐specific information but received high communication scores had patients that did not understand how to self‐manage their specific chronic illness effectively (Baker et al. 2005). Additionally, studies involving the self‐management of a respiratory chronic illness emphasised the importance of a therapeutic dialogue between patients and providers, while studies about diabetes communication tended to focus on how providers linking clinical measures to self‐management strategies helped support patients in self‐management (Piette et al. 2003; Peltola, Isotalus, and Åstedt‐Kurki 2018; Kruse et al. 2013; Kumar 2007; Heisler et al. 2007; Visse et al. 2010; Newcomb et al. 2010; Claramita et al. 2020). This finding speaks to the importance of providers not only demonstrating effective general communication but also delivering disease‐specific information.

## 11 Synthesis of Qualitative Studies

Qualitative studies coincide with quantitative studies and support the notion that patient–provider communication does influence patient self‐management of chronic illness. Patient perspectives in qualitative studies suggest that better patient–provider communication led to better self‐management. Data analysis from qualitative studies revealed the following themes: tailored information, patient engagement and provider collaboration, and knowledge translation.

### 11.1 Tailored Information

Patients preferred and found it more helpful to manage their chronic illnesses when providers delivered information specifically tailored and adapted for their illness (Ivynian, Newton, and DiGiacomo 2020). Patients were most satisfied when providers gave insights to how their disease process affected their ability to perform self‐management behaviours and their overall quality of life; this also improved patient knowledge and understanding of their chronic illness (Kumar 2007; Visse et al. 2010). When communication was not targeted to the patient's chronic illness, critical information was often missed and patients were left confused about matters concerning their illness (Newcomb et al. 2010). For example, patients who did not adhere to their asthma medication regimen reported not discussing their asthma medications with their provider and confusion surrounding these medications; on the reverse side, clinicians assumed that patients were taking their medication (Newcomb et al. 2010). Yet, when providers linked clinical measurements to patients' symptoms and explained the aetiology of patient's specific chronic illness, patients better understood how to and the importance of managing their chronic illness (Ivynian, Newton, and DiGiacomo 2020; Kruse et al. 2013). Being aware of and understanding the patient's social contexts influenced patient's decision making as it pertained to managing their chronic illness was also important. Australian immigrants reported feeling disempowered, anxious and confused about self‐management activities as a result of language and communication barriers (Jowsey, Gillespie, and Aspin 2011). These feelings were rooted in their inability to communicate with healthcare providers in their native language and the failure of health professionals to confirm their level of comprehension. Patients who had an instruction translated in their native language found them helpful to the management of their diabetes (Jowsey, Gillespie, and Aspin 2011).

### 11.2 Patient Engagement and Provider Collaboration

Patient involvement is crucial for patient‐centred care and in learning self‐management skills (Peltola, Isotalus, and Åstedt‐Kurki 2018; Kirk et al. 2023). When providers encouraged patients to share their experiences with illness as a story it provided a pathway for providers to offer practical support, advice and solutions for patients to manage their chronic illness (Kumar 2007; Visse et al. 2010). Promoting patient empowerment and engagement to improve awareness of their disease process helped patients prevent complications, and when patients were actively engaged in establishing goals for self‐management by communicating their preferences and values, they were able to achieve successful self‐management (Kumar 2007; Kirk et al. 2023; Claramita et al. 2020).

Considering a patient's cultural background is a key part of patient engagement and can have significant implications for patient self‐management and collaboration on a treatment plan. In Kumar's case study, cultural background and beliefs influenced the patient's management of her illness (Kumar 2007). It was through communication, with the nurse, that the clinical team discovered that the patient believed things were not in their control but ‘in God's hands,’ and they did not have the power or control to influence a health outcome. To address this, the patient and the nurse together developed a plan to improve the patient's belief and perceptions of being able to influence their own health outcomes. Culture was also suggested to shape expectations and communication behaviours regarding how messages are sensed as well as subsequent reactions during the clinical encounter (Peltola, Isotalus, and Åstedt‐Kurki 2018). Claramita et al. (2020) found that a culturally sensitive communication style of providers included in their study contributed to an increase in patient adherence to medication, physical exercise and regular monitoring of their chronic illness (Claramita et al. 2020).

Collaboration among providers and clinicians within the healthcare organisation can serve to facilitate patient–provider communication. When a nurse and a physician or advanced care provider are both present in the clinic environment, they can collaborate to address and meet the patient's needs (Kruse et al. 2013). For example, Kruse et al. (2013) found check‐listing, a rapid review of several indicators, treatments and practices specific to a chronic illness, to be a helpful strategy to ensure providers address important indicators of high‐quality care and have in‐depth conversation about patient self‐management (Kruse et al. 2013). This was achieved in collaboration with the nurse; while the nurse was responsible for addressing the medications, blood sugar testing and values, testing supplies, laboratory tests and eye examinations, the physician was allowed more time to focus on discussing a treatment plan and other issues conveyed by the nurse during their patient assessment.

### 11.3 Knowledge Translation

The goal of research is to embed evidence into practice to improve and inform healthcare outcomes and decision making (Barac et al. 2014). Knowledge translation is described in the literature as bridging the gap between research and practice, where healthcare providers use research evidence to inform their decision making. Sharing this knowledge with patients, applying it to clinical practice or using research to facilitate behaviour or practice change are all examples of knowledge translation (Barac et al. 2014). Knowledge translation is particularly important to chronic illness, and there is empirical evidence that suggests many patients with chronic illness desire information about their illness process and treatment options (Costello 2016; Manson 2010). Additionally, the failure to optimally use evidence in clinical practice results in inefficiencies and reduced quality of life for patients (Straus, Tetroe, and Graham 2011). Knowledge translation in this review concentrated on the way in which provider knowledge was communicated with the patient as how providers translate knowledge to the patient has considerable influence on patient self‐management of their chronic illness.

Face‐to‐face communication as well as written material and visual aids were reported to be helpful for patients to self‐manage their heart failure post hospitalisation (Ivynian, Newton, and DiGiacomo 2020). Making eye contact, sitting down fact to face and active listening were significant nonverbal communication behaviours perceived by the patient as willingness of the provider to communicate and creating experiences of care conducive to their own self‐management (Peltola, Isotalus, and Åstedt‐Kurki 2018). These nonverbal and verbal communication practices displayed by providers are central to interpersonal communication and are symbolic of a patient‐centred approach to knowledge translation (Peltola, Isotalus, and Åstedt‐Kurki 2018).

Communication style was found to play an important role in knowledge translation and patient self‐management. Provider's thoroughness of information provision rating (PCOM) and participatory decision making (PDM) were two styles of communication measured in association with patients reported diabetes self‐management among older adults (Heisler et al. 2007). Older adults were reported to prefer less of a PDM communication style and valued spending more time discussing self‐management and treatment options in detail, though both communication styles were associated with better self‐management of their diabetes (Heisler et al. 2007). Patients that had providers that communicated in a firm and authoritarian way experience a limited sense of control, empowerment and agency in the management of their diabetes (Jowsey, Gillespie, and Aspin 2011).

Lastly, when translating knowledge, it is important for providers to be cognizant of being too focused on the patient's illness and other disease‐specific quantitative measures as this can limit the transferability of information. This was evident in Kruse and colleagues' study on patient–provider communication and diabetes self‐management, where a difference in patient's and provider's views of measurable outcomes lead to a reduced interest in some self‐management activities (Kruse et al. 2013).

### 11.4 Synthesis of Findings From Quantitative and Qualitative Studies

By integrating the quantitative and qualitative findings of the articles included in this review, an overarching theme emerged: *adaptive interpersonal communication*. *Adaptive* references tailoring or adapting communication content to the individual needs of the patient, incorporating their needs and circumstances, and linking clinical measures to physical symptoms the patient experiences or outcomes of self‐management practices (Kirk et al. 2023; Newcomb et al. 2010). For example, a decrease in haemoglobin A1c may be a clinical measurement linked to patient the implementation of certain dietary practices or explaining to patient's experiencing shortness of breath when lying down and swelling in the legs they may have come from fluid volume overload, a physical manifestation of their heart failure (Ivynian, Newton, and DiGiacomo 2020; Kruse et al. 2013; Baker et al. 2005).

*Interpersonal* references the use of verbal and nonverbal communication to build a connection with patients and convey a willingness to communicate (Peltola, Isotalus, and Åstedt‐Kurki 2018; Kirk et al. 2023; Visse et al. 2010). It also references getting to know the patient outside of their illness, learning about their social circumstances and incorporating these matters into their communication about self‐management strategies. All of these strategies were shown to be significantly associated with improved patient self‐management of their chronic illness. While attributes of interpersonal communication were described differently for various patient populations, patients overall wanted to communicate with providers about their specific disease process and learn how to effectively manage their illness in a manner that was conducive to their learning and inclusive of their lifestyle preferences and values.

*Communication* references the entire knowledge translation process that takes place in patient–provider discussions about their chronic illness and how that translation of knowledge is perceived by the patient. Providers can no longer communicate with a sole focus on the illness and biomedical measures. Instead, providers must meet patients where they are and engage in meaningful and empathic conversations that help them get to know the patient as whole and offer supportive self‐management strategies. *Adaptive interpersonal communication* also considers the clinical encounter in which patient–provider communication occurs and acknowledges that there are health system and organisational barriers that may impede the knowledge translation process. Some of which can be addressed through acknowledgement and a willingness to change ingrained clinical practices. Part of this can be reflected in the collaboration among interdisciplinary providers and patients working together as partners in managing the care of the patient.

## 12 Discussion

Patient–provider communication is essential to patient self‐management of a chronic illness. Effective chronic illness self‐management requires following regimens for diet, medication and exercise (Heisler et al. 2007). These are not new insights, and there has been a substantive body of research examining skills and behaviours of chronic illness self‐management. There has not, however, been enough research to describe the influence of patient–provider communication on chronic illness self‐management. Of the studies that have addressed this phenomenon, opinions are mixed about the importance of patient–provider communication as it pertains to age groups, communication styles and how information should be delivered (Heisler et al. 2007). However, it is clear from this review that an *adaptive* and *interpersonal* approach to *communication* when developing a self‐management plan will lead to the most optimal outcomes. Given the importance of patient–provider communication for treatment outcomes, rigorous empirical evidence is needed to understand the effectiveness of different communication styles (Heisler et al. 2007). Additionally, we do not yet fully understand how patient–provider communication affects clinical outcomes and self‐management for minority and vulnerable populations (Heisler et al. 2007). This is an area ripe for future research given the current climate issues surrounding health equity. The limited reporting of race and ethnicity in the included studies further limits the ability to make inferences or describe the disparities that may exist in patient–provider communication and patient self‐management of a chronic illness.

The chronic illnesses included in this study are among the most common in the United States and provide significant implications for clinical care and chronic disease management. In this review, communication was evaluated in different ways including patient satisfaction with provider communication, perceived quality of patient–provider communication, provision of information and provider communication style (Peltola, Isotalus, and Åstedt‐Kurki 2018; Kruse et al. 2013; Visse et al. 2010). Additionally, various analysis methods and study designs were also employed in the studies included in this review. This is particularly important as we continue to build upon current knowledge to develop interventions that improve communication between patients and providers and patient self‐management of chronic illness. Many of the studies included in this review were of good methodological quality as it pertains to the approach to answer the research question, data collection and analysis methods, interpretation and coherence between all three of these factors. Relational communication, the Chronic Care Model, Orem's self‐care deficit theory and patient‐centred care (PCC) are among the guiding frameworks, models, theories or concepts for many of the studies included in this review.

While this review focused on patient self‐management of chronic illness as an outcome, specific measured outcomes included clinical measures (blood pressure, haemoglobin A1c), symptom and self‐efficacy scales, and medication adherence. However, it should not be ignored that to improve health outcomes, factors such as healthcare access and quality of care must also be addressed (Baker et al. 2005). A patient‐centred approach is often aligned with the delivery of high‐quality care. It is traditionally described as a philosophical orientation and defined through the relational communication behaviours between the patient and their healthcare providers (Peltola, Isotalus, and Åstedt‐Kurki 2018; Gluyas 2015). It is the responsibility of the provider, as opposed to the patient, to place experiences, values, needs and preferences of the patient at the centre of planning, co‐coordination and delivery of care (Peltola, Isotalus, and Åstedt‐Kurki 2018; Gluyas 2015). In this review, we found that when providers engage and communicate with patients respectfully and ensure their understanding, patients were in turn motivated to improve self‐management behaviours (Peltola, Isotalus, and Åstedt‐Kurki 2018). Information concerning patient self‐management of their chronic illness is more powerful when it is linked and adapted directly to patients' specific preferences, circumstances and behaviours (Heisler et al. 2007). It is this link that ultimately drives the improvement of clinical outcomes because patients are presented with realistic and practical solutions to apply to their everyday life. Providers should give considerable attention to the patient's priorities, obstacles to self‐management and strategies for overcoming obstacles. One way this can be done is by translating knowledge of how some clinical measures such as blood pressure and glycosylated haemoglobin are connected to how poorly or well a patient may feel (Hong, Gonzalez‐Reyes, and Pluye 2018). Additionally, when sharing knowledge with patients, providers should opt to use plain language and develop a clear and concise action plan, in collaboration with the patient, to manage their symptoms and chronic illness (Ivynian, Newton, and DiGiacomo 2020). It is worth noting that adaptive interpersonal communication could be a consequence of good self‐management rather than the other way around. Patients that are naturally proactive in advocating for themselves and managing their health or chronic illness may embody or demonstrate interpersonal communication skills with their healthcare providers. These patients, who take the initiative to educate themselves about their condition, may share valuable information with their providers regarding their circumstances and lifestyle preferences. This proactive approach then facilitates communication with healthcare providers.

Findings from this review have implications for individuals, health systems and providers to guide development and improvement of communication skills. Nurses play a crucial role in patient–provider communication, particularly in the clinical setting (Kourkouta and Papathanasiou 2014). Previous study has found that quality communication between patients and nurses has a significant influence in fostering compassionate relationships, patient satisfaction and improved care outcomes (Kwame and Petrucka 2021). Yet, few studies have examined the influence of nurse–patient communication on chronic disease outcome, with studies in this primarily focusing on physician impact. Given the increasingly critical role of nurses in care management, future research should investigate the impact of nurse's communication on patient self‐management (Hassmiller and Wakefield 2022). Uncovering this impact not only contributes to a more holistic patient‐centred approach to chronic disease management but also facilitates the development of communication strategies and educational curriculum content aimed at implementing effective training methods for all healthcare professional caring for this population. Additionally, many of the studies included in this review did not report on the provider type (i.e., nurse, nurse practitioner, physician) or their demographic information. Future studies on patient–provider communication should include demographics on both patients and providers.

Organisational and health system culture also play a key role in patient–provider communication, which indirectly affects patients and their ability to self‐manage. In consideration of multiple competing demands during short office visits, it is imperative for providers to tailor communication to most effectively support patient's self‐management of their chronic illness (Heisler et al. 2007). However, when providers face barriers such as time constraints, poor reimbursement and difficulty changing habitual clinical practices, it is difficult for providers to effectively promote self‐management to patients (Kruse et al. 2013). Health systems and organisations should work to structure a clinical environment that intentionally supports knowledge translation between patients and providers and encourages patient engagement in decision making (Heisler et al. 2007). Part of working towards this goal may be adopting an interdisciplinary approach, where health professionals from different disciplines, along with the patient, work collaboratively as a team to optimise patient visits and provide self‐management support (McDonald, Jayasuriya, and Harris 2012; Liddy et al. 2019).

The relationship between patient race, language, culture in the context of chronic illness and patient–provider communication is poorly understood, deeming this phenomenon worthy of further investigation. A lack of culturally competent patient–provider communication can serve as a barrier to delivering complex health information to patients about managing their chronic illness (Brown et al. 2016). Additionally, studies that employ a healthcare equity lens or framework may lend support for investigations that consider if patient expectations or perceptions of provider communication differ across race, age, gender and other sociodemographic variables. The lack of consideration of healthcare equity in the studies included in this review places boundaries for interpretation of findings. Jowsey and colleagues (Jowsey, Gillespie, and Aspin 2011) focused on the context of language and culture when communicating self‐management strategies to Australian immigrants and emphasised the importance of the healthcare system investing in resources to support this vulnerable population. Given the current patterns of migration in the United States, strengthening self‐management strategies that address the needs of migrants is critical to supporting their self‐management of chronic illness.

Patients were variably successful in developing the skills needed to self‐manage their chronic illness (Piette et al. 2003; Peltola, Isotalus, and Åstedt‐Kurki 2018; Kruse et al. 2013; Kumar 2007; Claramita et al. 2020). In fact, patients often understand what they are supposed to do but are frequently prevented from being able to do so because of external demands, such as caring for other family members or work. Information concerning patient self‐management of their chronic illness is more powerful when it is linked and adapted directly to patients' specific preferences, circumstances and behaviours (Heisler et al. 2007). It is this link that reflects the *adaptive* component of the overarching theme in this review and ultimately drives the improvement of clinical outcomes because patients are presented with realistic and practical solutions to apply to their everyday life. Lack of communication surrounding the day‐to‐day management of chronic illness can lead to unnecessary changes in a patient's plan of care. This was evident in Newcomb et al.'s (2010) study on asthma management when a failure of the provider to discuss the patient's home management of asthma and adherence to their medication regimen led to an increase in medication dosage, which may have not been warranted had the provider known the patient had not been following their medication regimen (Kumar 2007). When providers consider patients experiences, values and needs, they also should take into consideration the social context of the patient, particularly when developing a plan for self‐management (Peltola, Isotalus, and Åstedt‐Kurki 2018; Kruse et al. 2013; Visse et al. 2010). Providers should probe patients to share their life commitments, daily activities and various social circumstances. This helps patients and providers collaborate to develop self‐management strategies that work best for the patient (Visse et al. 2010). Failure to consider patient's social circumstances results in missed opportunities for providers to educate patients on the significant role their home and social environment plays in their ability to effectively self‐manage their chronic illness. More importantly, when providers do not engage the patient in discussions about their social circumstances, patients often abandon general self‐management strategies they perceive as ineffective because they are not realistically applicable to their life circumstances (Kumar 2007; Newcomb et al. 2010).

### 12.1 Limitations

There are limitations to this review, as only studies published in English and available in four databases (CINAHL, Google Scholar, EMBASE, PubMed) were included. Although the search strategy was developed in collaboration with a librarian and varied combinations of search terms were employed, some relevant literature may be missing. While there were a variety of chronic conditions included in this review, the findings cannot be generalised for all chronic conditions. Additionally, none of the studies included in this review included any health equity concepts, and few reported the race or ethnicity of the patient population, making generalisability and application to vulnerable and marginalised populations difficult. Moreover, demographic and provider type descriptions were missing from many of the articles, thereby limiting the ability to characterise the providers included in this review. Lastly, articles were restricted to patient–provider communication and self‐management only; articles that discuss the patient–provider relationship, rather than just communication, may have yielded additional findings not included in this review.

## 13 Conclusion

Findings from this review remind the healthcare community of the importance and significance patient–provider communication has on patient self‐management of chronic illness. The findings of this review have significant implications for practice and policy. Healthcare providers should receive training and ongoing professional development in deploying effective communication techniques that are tailored to individual patients' needs. Interventions that focus on implementing communication strategies described in this review into personalised action plans and other self‐help materials are needed. Moreover, these interventions can work to improve patient understanding and engagement in their care. The implementation of future interventions should also include continuous evaluation and feedback on communication strategies and assess the effectiveness of interventions to make necessary improvements.

Additionally, there are systemic challenges and barriers that may hinder the implementation of communication strategies described in this review. These include limited consultation time, inadequate resources and fragmented healthcare system. Researchers should build upon these findings to identify the proper support and resources needed for providers to implement tailored and adaptive communication approaches. Policymakers should leverage the insights from this review to inform the development of clinical environments that are more conducive to the time and effort needed for providers to have thorough discussions about patient's conditions, treatment options and preferences, and context‐appropriate self‐management strategies. Lastly, patients with chronic illnesses often require services from multiple parts of the healthcare system, potentially resulting in fragmented care. Future research should examine the impact of fragmented care on patient–provider communication and patient self‐management of their chronic illnesses, with the aim of developing integrated approaches that enhance communication and improve self‐management outcomes.

## Conflicts of Interest

The authors declare no conflicts of interest.

### Peer Review

The peer review history for this article is available at https://www.webofscience.com/api/gateway/wos/peer‐review/10.1111/jan.16492.

## Data Availability

Data utilised in the submitted manuscript have been lawfully acquired in accordance with The Nagoya Protocol on Access to Genetic Resources and the Fair and Equitable Sharing of Benefits Arising from Their Utilization to the Convention on Biological Diversity.
